# Decision Tree Analysis: A Retrospective Analysis of Postoperative Recurrence of Adhesions in Patients with Moderate-to-Severe Intrauterine

**DOI:** 10.1155/2019/7391965

**Published:** 2019-12-12

**Authors:** Ru Zhu, Hua Duan, Sha Wang, Lu Gan, Qian Xu, Jinjiao Li

**Affiliations:** ^1^Department of Minimally Invasive Gynecology, Beijing Obstetrics and Gynecology Hospital, Capital Medical University, Beijing 100006, China; ^2^Department of Obstetrics and Gynecology, Anqing Hospital Affiliated to Anhui Medical University, Anqing 246003, China

## Abstract

**Objective:**

To establish and validate a decision tree model to predict the recurrence of intrauterine adhesions (IUAs) in patients after separation of moderate-to-severe IUAs.

**Design:**

A retrospective study.

**Setting:**

A tertiary hysteroscopic center at a teaching hospital.

**Population:**

Patients were retrospectively selected who had undergone hysteroscopic adhesion separation surgery for treatment of moderate-to-severe IUAs.

**Interventions:**

Hysteroscopic adhesion separation surgery and second-look hysteroscopy 3 months later.

**Measurements and Main Results:**

Patients' demographics, clinical indicators, and hysteroscopy data were collected from the electronic database of the hospital. The patients were randomly apportioned to either a training or testing set (332 and 142 patients, respectively). A decision tree model of adhesion recurrence was established with a classification and regression tree algorithm and validated with reference to a multivariate logistic regression model. The decision tree model was constructed based on the training set. The classification node variables were the risk factors for recurrence of IUAs: American Fertility Society score (root node variable), isolation barrier, endometrial thickness, tubal opening, uterine volume, and menstrual volume. The accuracies of the decision tree model and multivariate logistic regression analysis model were 75.35% and 76.06%, respectively, and areas under the receiver operating characteristic curve were 0.763 (95% CI 0.681–0.846) and 0.785 (95% CI 0.702–0.868).

**Conclusions:**

The decision tree model can readily predict the recurrence of IUAs and provides a new theoretical basis upon which clinicians can make appropriate clinical decisions.

## 1. Introduction

Intrauterine adhesions (IUAs) occur due to damage to the basal layer of the endometrium from various causes and disrupt the uterine anatomy [1]. Intrauterine adhesions occur in 1.7% to 45.5% of women with various conditions or after various uterine surgeries [2, 3]. The rate is 19.1% after dilatation and curettage, and 42% of these are moderate-to-severe [4]. The main manifestations of IUAs are pain, menstrual abnormalities, and reproductive disorders such as infertility, repeated loss of pregnancies, premature delivery, and obstetric complications [5].

The standard treatment for IUAs is lysis under direct hysteroscopic visualization [6]. However, after severe IUAs, the recurrence rate is as high as 20% to 62.5% [7]. Therefore, IUAs severely affect women's menstrual physiology, reproductive function, and mental health.

As standardized diagnoses and treatment of IUAs have become more common, the importance of the first hysteroscopic lysis has been increasingly emphasized. The hysteroscopic lysis should avoid further damage to the residual endometrium and the burden of multiple operations on patients [8, 9]. At present, there is no practical method to predict the recurrence of IUAs after a separation procedure. The inadequacy of preoperative evaluations may increase the rate of repetitive surgery and surgical complications [10]. Therefore, a method is urgently needed for predicting the recurrence of uterine adhesions after separation.

Multivariate logistic regression analyses are used to predict clinical outcomes and identify risk factors. However, they are difficult to implement and explain, especially for clinicians without training in statistics, and therefore are difficult to apply and promote in clinical practice [11]. Alternatively, a decision tree model can determine the attribute variables that deliver the most meaningful information for classification and prediction. The results are displayed in a tree-like structure, which greatly facilitates the recognition and application of the results by clinicians [12–15].

In the present study, a decision tree model was constructed and validated for the recurrence of IUAs. Complete patient data collected previously were incorporated, and the relevant factors that may affect the recurrence of IUAs were determined [16, 17]. For reference, a multivariate logistic regression analysis was conducted, and the two models were compared. The decision tree model screens for factors affecting the recurrence of IUAs and simultaneously can preoperatively predict recurrence after hysteroscopic lysis. Thus, the decision tree model provides a theoretical basis for clinicians to evaluate the therapeutic effect and select appropriate treatment strategies.

## 2. Methods

This study was approved by the Medical Ethics Committee of Beijing Obstetrics and Gynecology Hospital.

### 2.1. Patients

Patients were retrospectively selected, who had undergone hysteroscopic adhesion separation surgery for treatment of moderate-to-severe IUAs from January 2013 to December 2017 at Beijing Obstetrics and Gynecology Hospital Affiliated with Capital Medical University. The demographic, clinical history, imaging, and hysteroscopy evaluation data were collected. The center is a tertiary medical institution, in which almost 1000 patients receive hysteroscopy surgery annually. Patients with mild IUAs are treated in the outpatient clinic, and most of these lack a second exploration by hysteroscopy after surgery. These patients were not included in this study.

All the subjects conformed to the following criteria: outpatient hysteroscopy diagnosis of moderate-to-severe IUAs (American Fertility Society score ≥5) [18]; the prior menstrual cycle was regular; and normal sex hormone profile. Patients with any of the following were excluded from this study: the surgery failed to restore normal intrauterine anatomy; no second hysteroscopy was performed within 3 months after surgery; or the presence of endometrial lesions or uterine malformation.

**Figure 1:**
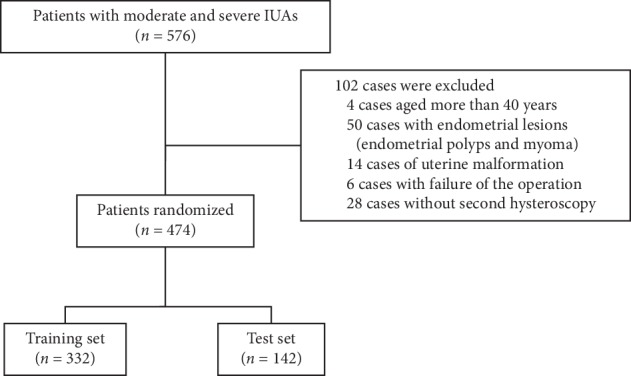
Schematic of patient screening.

A database for the present study was set up with complete information. Data were randomly apportioned to a training set or a test set at a group size ratio of 7 : 3 (Figure 1).

### 2.2. Predictive Measures

A unified medical report form was developed for all patients. The relevant data were collected from an electronic inpatient database, including the following: age; number of pregnancies/childbirths; pregnancy loss; uterine cavity operations; hysteroscopic lysis; organic lesions related to the reproductive system; menstrual pattern; causes of uterine adhesions; and ultrasound measurements of uterine volume and endometrial thickness. Preoperative menstrual patterns were recorded as amenorrhea; ≤25% of normal menstruation; 25% to 50% of normal; or ≥50% of normal. Causes of uterine adhesions were considered as factors related to pregnancy (first-trimester termination of pregnancy and second or third trimester termination of pregnancy) or factors related to nonpregnancy.

All patients underwent vaginal color Doppler ultrasound (GE E8). Patients with hypomenorrhea were selected to undergo ultrasonography at midcycle. Patients with amenorrhea were examined at any time. Endometrial thickness was measured from the echogenic interface at the junction of endometrium and myometrium, at the level of the maximum anteroposterior diameter in the sagittal plane. The length, width, and thickness of the uterus were measured. The length of the uterus was from the fundus uteri to internal os of the cervix in the sagittal plane. The width and thickness of the uterus were measured in the coronal and sagittal planes, respectively. The volume of the uterus was measured as *V*, cm^3^ = length × width × thickness × 0.523.

During the preoperative hysteroscopy evaluation, the IUAs were scored in accordance with the American Fertility Association [18], as follows: mild, 1–4; moderate, 5–8; or severe, 9–12. At the same time, the types of IUA, the depth of the uterine cavity, and the closure of the uterine horn and tubal ostia (visible or invisible) were recorded by a full-time experienced hysteroscopy evaluator.

There were 3 types of isolation barriers: heart-shaped copper intrauterine device (IUD), Foley balloon, and intrauterine suitable balloon [19].

### 2.3. Outcome Index Measures at Follow-up

The patients were followed up in the outpatient clinic 3 months after the initial operation. A hysteroscopy was performed with a 4.5 mm hysteroscope, using normal saline as the perfusion fluid. The procedure was performed by a full-time experienced hysteroscopy evaluator in the outpatient clinic.

### 2.4. Operative Procedures and Postoperative Preventive Measures

Hysteroscopic lysis was performed by an experienced endoscopic surgeon. All patients were under general anesthesia. The surgical equipment and instruments were as follows: Olympus S70 operation hysteroscope series equipment; operation hysteroscope; and matched 27Fr passive continuous perfusion bipolar electroscope. The cutting and coagulating power were set at 320 W and 160 W, respectively. The perfusion medium was normal saline. Tracheal intubation plus venous-combined general anesthesia was used for surgical anesthesia. We pretreated with 200 to 400 mcg of vaginal misoprostol, 12 to 24 hours prior to hysteroscopy. Hysteroscopic lysis was guided by transabdominal ultrasonography.

We used needle electrodes to cut the adhesion tissue and ring electrodes to resect the dense scar tissue. During the entire operation, we took special care to identify and protect the remaining normal intima tissue. Successful separation of adhesions should restore the normal intrauterine anatomy without adhesions. Uterine horns and tubal ostia were visible or invisible.

After the separation of IUAs, different isolation barriers were placed in the uterine cavity. The choice of the isolation barrier depended primarily on the preferences of the surgeons. An intrauterine suitable balloon (Patent number: 201420679083.7) was used after surgical separation [19]. Firstly, the gas in the intrauterine suitable balloon was aspirated to exert a negative pressure. It was then wrapped around the lumen and rotated along the cervical canal into the endometrial cavity. Subsequently, 3-4 mL saline was injected into the balloon, which was fully expanded in the intrauterine cavity. The balloon catheter was connected to the drainage bag device. The intrauterine suitable balloon was removed 5 to 7 days after the operation, by aspirating the saline and withdrawing the balloon. The method of placing and pulling out the Foley balloon was similar to that of the intrauterine suitable balloon. A heart-shaped copper intrauterine device (IUD) was inserted and removed 3 months later during the follow-up hysteroscopy. Complications were recorded.

All patients were administered the same 3 hormonally controlled cycles on the second day after surgery, as follows. For each cycle, oral administration of 4 mg/d estradiol valerate tablets (Progynova; Bayer; Delpharm Lille S.A.S) for 21 days and 20 mg/d dydrogesterone tablets (Abbott Biologicals) for the next 10 days was performed. Antibiotic therapy (Sichuan Hexin Pharmaceutical, Sichuan, China) was routinely administered to reduce the risk of infection, for 7 days [20].

### 2.5. Statistical Analysis

The original data included 18 variables, and all variables were uniformly quantified and encoded (Table 1). Three months after surgery, the recurrence of adhesions found by hysteroscopy was taken as the outcome index, and 17 other related factors were taken as the predictive indices. The attribute variables or categories of each influencing factor were described. A Random-Forest software package in R language (http://www.r-project.org) was used to rank the features of independent variables, according to the mean reduction accuracy index. The top 10 important feature structures were selected, and the decision tree model was established by CART (classification and regression tree) algorithm [21]. We used 70% of the original data as a training set to train the decision tree, and the remaining 30% as a testing set to verify the decision tree. The measure used to split nodes was the Gini index, and pruning was used to avoid overfitting the model.

A multivariate logistic regression model (SPSS 23.0) was constructed using the first 10 predictive variables of feature ranking. *P* < 0.05 was considered statistically significant.

## 3. Results

Initially included in this study were 576 patients who had moderate-to-severe IUAs within the previous five years (Figure 1). The 102 excluded patients comprised 4 and 6 patients due to older age and unsuccessful surgery, respectively; 28 patients lost to follow-up; and 64 with intrauterine or uterine lesions (polyps, myoma, endometrial lesions, adenomyosis, uniangular uterus, or uterus septum). Finally, 474 patients (aged 31.5 ± 4.09 years) with complete data were included in the study analysis. Isolation barriers were used in all patients after surgery. A heart-shaped copper intrauterine device (IUD), Foley balloon, and intrauterine suitable balloon accounted for 57.2%, 21.3%, and 21.5%, respectively.

In this study, moderate-to-severe IUAs were mainly caused by pregnancy-related curettage (92.4%; 438/474). The overall recurrence rate was 32.3% (153/474). The complete data of 474 patients were randomly divided into a training set (332 patients) and a test set (142 patients). There was no significant difference between the two groups with regard to predictive and outcome indicators. The recurrence rates of the training and test set groups were 32.5% (108/332) and 31.7% (45/142), respectively.

Based on the results of feature ranking of the average accuracy reduction index (Table 2), the top ten variables were used to construct the decision tree with training set samples. In the decision tree, the American Fertility Society (AFS) score, isolation barrier, endometrial thickness, tubal opening, uterine volume, and menstrual pattern are the classification node variables for recurrence of IUAs; the AFS score is the root node variable.

**Figure 2:**
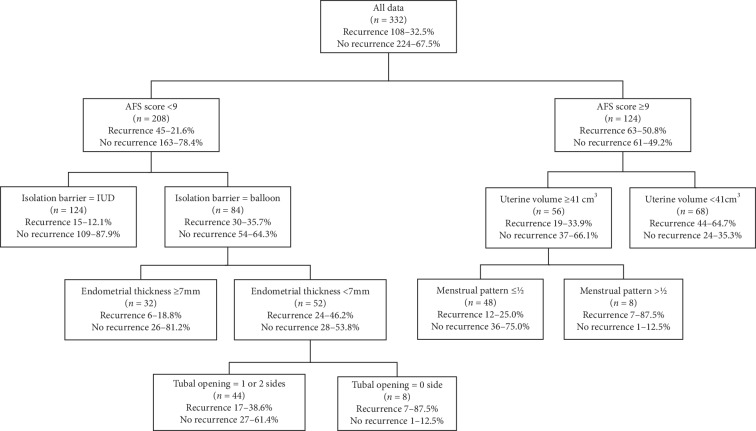
Decision tree model for recurrence after IUA surgery. Balloon = intrauterine suitable balloon or Foley balloon.

The recurrence rate of adhesions of patients with an AFS score ≥9 points (50.8%) was significantly higher than that of patients with an AFS score <9 points (21.6%; Figure 2). For patients with an AFS score ≥9 points, the rate of adhesion recurrence was greater in those with uterine volume <41 cm^3^ (64.7%) compared with those with uterine volume ≥41 cm^3^ (33.9%). Among the patients with AFS score <9 points, the rate of adhesion recurrence in those with a heart-shaped copper IUD (12.1%) was lower than that of patients with a balloon device (35.7%).

**Figure 3:**
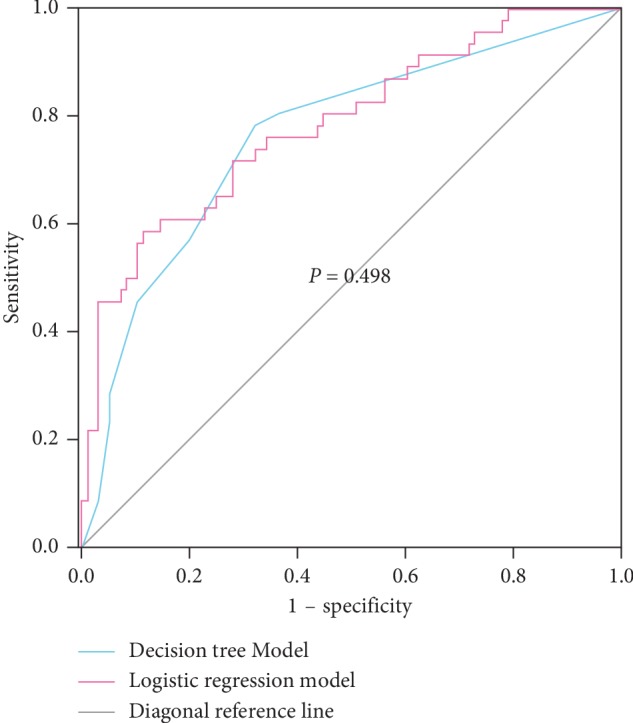
Area under the receiver operating characteristic curve of the decision tree model and multivariate logistic regression model.

The predictive accuracy of the decision tree model is 75.35%, and the area under the receiver operating curve is 0.763 (95% CI 0.681–0.846; Figure 3). The predictive accuracy of the multivariate logistic regression analysis model is 76.06%, and the area under the receiver operating curve is 0.785 (95% CI 0.702–0.868). The effectiveness of the two prediction models is not statistically significant (*P*=0.498) [22]. The independent risk factors for recurrence of IUAs were the following: isolation barrier; etiological type; endometrial thickness; and tubal opening (Table 3).

## 4. Discussion

This study successfully established and validated a decision tree model for postoperative adhesions in patients with IUAs. The accuracy of the model was 75.35%, which was comparable to the accuracy of the multivariate logistic regression analysis model, and has high predictive value. The structure of the decision tree model clearly delineates the decision-making process, the probability of recurrence of adhesion after surgery, and the risk factors that correlated with recurrence after surgery. It not only provides a theoretical basis for making clinical decisions, but also facilitates communications between doctors and patients.

The model shows that an AFS score ≥9 is the root node of recurrence of IUAs. Thus, the AFS score is the most important factor affecting the recurrence of IUAs. The recurrence of IUAs is also closely related to the degree of IUAs. This is consistent with previous studies [8, 23].

When the AFS score was <9 points, the isolation barrier in the decision tree was second in order only to the AFS score. This indicates that recurrence is affected by different isolation barriers. When the preoperative AFS score was <9 points, the postoperative preventive effect of the heart-shaped copper IUD was better than that of the balloons.

One study showed that the Foley catheter, compared with the IUD, is a safer and more effective adjunctive method of treatment for intrauterine adhesions, but there was no effective method to evaluate the recurrence of adhesions [24]. A recent randomized controlled study determined that the heart-shaped copper IUD and heart-shaped intrauterine balloon were equally effective for preventing adhesion recurrence [25]. The differences may be due to the different shapes of intrauterine devices. The heart-shaped copper IUD is more effective for isolating peripheral adhesions [2, 25]. Alternatively, differences may be related to the degree of adhesion.

In the present study, among the patients with an AFS score <9 points, the recurrence rate of adhesions of those with a heart-shaped copper IUD was much lower than that of patients with a balloon device. This suggests that the effect of using a heart-shaped copper IUD after surgery was better than that of the balloon device, mainly for patients with moderate adhesion. Previous studies found that the intrauterine suitable balloon was superior to the Foley balloon for preventing the recurrence of severe IUAs [19]. In the present decision tree model, there is no difference between the two balloons in preventing recurrence. Perhaps, because the decision tree model is based on local optimization and the number of IUD samples is large, the effect of the IUD was obviously higher than the two balloon devices.

When the AFS was ≥9, the isolation barrier was no longer a categorized node variable affecting the outcome. We think this may be due to two main factors. First, although the separation of IUAs can restore the uterine anatomy of patients with severe IUAs, it is difficult to repair the wound and restore the function of the endometrium, the recurrence rate of adhesion is high, and the effect of postoperative adjuvant measures on preventing recurrence of adhesion is limited. Secondly, the decision tree was pruned to simplify decision making. There is still controversy about what preventive measures should be used after surgery, and further randomized controlled trials are needed [26].

In patients with an AFS <9 and using a balloon as isolation barrier after surgery, endometrial thickness is a classified node variable, and an endometrial thickness <7 mm is a high-risk factor for recurrence of adhesion after surgery. Previous studies have also suggested that thickness is closely associated with recurrence of uterine adhesions and pregnancy outcomes [27, 28].

Another risk factor for recurrence of IUAs is tubal ostium. This is consistent with the relevant classification of IUAs. The opening of the fallopian tube not only reflects the degree of adhesions, but also is related to the outcome of pregnancy after surgery. Many international classification standards of IUAs regard this as a classification index [6, 29, 30].

In patients with AFS ≥9, the decision tree model also showed that uterine volume and volume of menstrual flow was associated with recurrence of IUAs. That is, the smaller the uterus, the higher the recurrence rate, as well as the smaller the uterus, the more vulnerable it is to injury, and the more serious the injury.

The menstrual pattern was assessed according to patients' self reports. The decision tree algorithm found that one-half of the normal amount of menstruation was a categorized variable. To some extent, the menstrual pattern reflects the degree of adhesion and residual normal functional endometrial area, which can be used as an index to predict the recurrence of uterine adhesions after surgery [6, 18]. The results of the present study found that recurrence of adhesions was more common when menstrual volume was higher. This may be related to different characteristics of the patients, and the decision tree mainly considers the principle of local optimality. Because the number of patients is relatively small, further data are needed. In future studies, we will assess the menstrual blood loss using a menstrual pictogram.

So far, there is no model for predicting recurrence after IUA separation. In the present study, predictive indicators were quantified before the decision tree was built. The decision tree model was simplified by feature extraction, making it easier to apply in clinical practice [31]. At the same time, a multivariate logistic regression model was established. The predictive accuracy of the two models was similar, but the decision tree model is easier to implement, understand, and interpret [13].

The decision tree model has unique advantages in predicting recurrence of IUAs after surgery. Firstly, it reveals the importance of related factors in the recurrence of adhesions. The closer it is to the root node, the greater is the influence of classification variables on outcomes. It simultaneously categorizes patients with different characteristics, which is more important than only analyzing which variables affect the outcomes. The decision tree model has more guiding and practical significance [13].

Secondly, the tree structure of the decision tree model can clearly show the recurrence rate of patients with different characteristics after surgery. Clinicians can know the particular probability of recurrence of adhesions before surgery, then choose whether to operate or not according to the local medical technology and the patient's condition, and avoid blindly choosing certain, excessive, and ineffective treatments. Reasonable selection of antiadhesion measures can also help reduce the recurrence of adhesions.

Thirdly, the decision tree model also shows the interaction between variables, specifically analyzing the way a variable behaves within the subgroup. The model helps to identify high-risk factors for the formation of adhesions or poor prognosis after treatment. This can aid clinical decisions to prevent the formation of initial adhesions, such as avoiding unnecessary curettage after abortion or repeated curettage and using measures to promote endometrial repair after curettage [1, 3, 32, 33].

Mechanical instruments are usually used when adhesiolysis is performed, as the use of energy may damage the endometrium and the zona basalis, leading to adhesions. The use of energy may also influence the rate of recurrent adhesion, which was quite high in the current study, and has the potential to affect reproductive outcomes. Therefore, we used mechanical instruments.

The decision tree of the present study is limited with regard to stability of results and local hierarchical analysis [13, 34]. The stability and predictive accuracy of the decision tree model are closely related to the data of the training set. More data will be included at a later stage, including different surgical methods, surgical instruments, isolation measures, and methods to promote the growth of endometrium. Another limitation is the retrospective nature of the study, with its inherent bias. Continuous updating of the model will benefit its clinical application.

## 5. Conclusions

The decision tree model for recurrence of IUAs after surgery can readily predict the recurrence of IUAs and help doctors to make preoperative decisions and choose appropriate prevention programs after surgery. Future data will improve the stability and predictive value of the decision tree model.

## Figures and Tables

**Table 1:** Attribute variables or category descriptions of factors influencing the recurrence of intrauterine adhesions.

	Data type	Encoding
Recurrence of adhesions	Binary classification	No; yes

Age (y)	Continuous variable	—
Times of pregnancy	Frequency	—
Childbirth	Binary classification	No; yes
Menstrual pattern	Quadruple classification	*z* = 0, *a* ≤ 1/4, *b* = 1/4–1/2, *c* ≥ 1/2

Etiology	Tripartite classification	*a* = termination of early pregnancy; *b* = termination of mid-late pregnancy; *c* = nonpregnancy-related factors

Pregnancies lost (*n*)	Tripartite classification	*z* = 0, *a* = 1, *b* ≥ 2
Intrauterine operations (*n*)	Frequency	—

Previous TCRA	Binary classification	No; yes
Endometrial thickness (mm)	Tripartite classification	*a* ≤ 3 mm, *b* = 4–6 mm, *c* = ≥7 mm

Uterine volume (cm^3^)	Continuous variable	—
AFS score	Continuous variable	—

Degree of adhesion	Two classifications	*a* = moderate intrauterine adhesions, *b* = severe intrauterine adhesions

Adhesion type	Two classifications	*a* = mixed type, *b* = peripheral type
Intrauterine depth	Continuous variable	—

Uterine horn closure	Tripartite classification	*z* = 0 side, *a* = 1 side, *b* = 2 sides
Tubal ostia	Tripartite classification	*z* = 0 side, *a* = 1 side, *b* = 2 sides

Isolation barrier	Tripartite classification	*a* = Foley balloon, *b* = intrauterine suitable balloon, *c* = intrauterine contraceptive device

**Table 2:** Reduction of mean accuracy by sorting order.

	Variable	Decreased accuracy
1	Isolation barrier	42.17

2	AFS score	40.36

3	Tubal ostia	34.16

4	TCRA	25.66

5	Degree of adhesion	24.28

6	Uterine volume (cm^3^)	22.54

7	Adhesion type	21.71

8	Endometrial thickness (mm)	16.40

9	Etiological type	15.47

10	Menstrual pattern	14.19

11	Uterine horn closure	5.43

12	Times of pregnancy	1.84

13	Intrauterine depth	1.26

14	Age (y)	1.07

15	Lost pregnancies (*n*)	–1.25

16	Childbirth	–1.80

17	Intrauterine operations (*n*)	–4.10

**Table 3:** Multivariate logistic regression analysis.

	*β*	SE	Wald	*P*	OR (95% CI)
Isolation barrier	–0.603	0.138	18.939	<0.001^*∗*^	0.547 (0.417–0.718)
AFS score	0.170	0.127	1.809	0.179	1.186 (0.925–1.520)
Tubal ostia	–0.306	0.148	4.251	0.039^*∗*^	0.736 (0.550–0.985)
TCRA	0.541	0.298	3.300	0.069	1.718 (0.958–3.080)
Degree of adhesion	0.115	0.452	0.065	0.799	1.122 (0.463–2.723)
Uterine volume	–0.011	0.007	2.756	0.097	0.989 (0.976–1.002)
Adhesion type	–0.328	0.265	1.533	0.216	0.720 (0.428–1.211)
Endometrial thickness	–0.426	0.184	5.334	0.021^*∗*^	0.653 (0.455–0.938)
Etiological type	0.515	0.182	7.985	0.005^*∗*^	1.674 (1.171–2.393)
Menstrual pattern	–0.108	0.133	0.658	0.417	0.898 (0.692–1.165)

Note: *P* < 0.05 was considered statistically significant.

## Data Availability

The data used to support the findings of this study are available from the corresponding author upon request.
